# Pan-Cancer Analysis Reveals Genomic and Clinical Characteristics of TRPV Channel-Related Genes

**DOI:** 10.3389/fonc.2022.813100

**Published:** 2022-01-31

**Authors:** Xiaoxuan Wang, Guanghao Li, Yidan Zhang, Lanfang Li, Lihua Qiu, Zhengzi Qian, Shiyong Zhou, Xianhuo Wang, Qiang Li, Huilai Zhang

**Affiliations:** ^1^ Department of Lymphoma, Tianjin Medical University Cancer Institute and Hospital, National Clinical Research Center of Cancer, Key Laboratory of Cancer Prevention and Therapy, Tianjin’s Clinical Research Center for Cancer, Sino-US Center for Lymphoma and Leukemia Research, Tianjin, China; ^2^ Department of Hepatobiliary Cancer, Tianjin Medical University Cancer Institute and Hospital, National Clinical Research Center for Cancer, Key Laboratory of Cancer Prevention and Therapy, Tianjin’s Clinical Research Center for Cancer, Tianjin, China

**Keywords:** pan-cancer, methylation, genomics, prognosis, TRPV channel-related genes

## Abstract

**Background:**

Transient Receptor Potential channels (TRPs), a class of ion channels, were first described two decades ago. Many TRP family members are major participants in nociception and integration of heat and pain signals. Recent studies have revealed that subfamilies of this channel, such as members of transient receptor potential vanilloid (TRPV) channels, play important roles in breast, ovarian, prostate, and pancreatic cancers.

**Methods:**

We performed a comprehensive analysis of TRPVs in 9125 tumor samples of 33 cancer types using multi-omics data extracted from The Cancer Genome Atlas (TCGA). We identified differences in mRNA expression in a pan-cancer analysis, and the genomic characteristics of single nucleotide variations, copy number variations, methylation features, and miRNA–mRNA interactions using data from TCGA. Finally, we evaluated the sensitivity and resistance to drugs targeting TRPV channel-related genes using the Cancer Therapeutics Response Portal (CTRP) and the Genomics of Drug Sensitivity in Cancer (GDSC) database. Finally, we validated the drug sensitive data and the importance of TRPV6 in two cancer cell lines using q-PCR assay, CCK8 assay, EdU assay and scratch assay.

**Results:**

Extensive genetic alterations in TRPV channel-related genes and differences in gene expression were associated with the activity of cancer marker-related pathways. TRPV channel-related genes can be used as prognostic biomarkers. Several potential drugs, such as lapatinib, that may target TRPV channel-related genes were identified by mining the genomics of drug sensitivity.

**Conclusion:**

This study revealed the genomic changes and clinical characteristics of TRPV channel-related regulatory factors in 33 types of tumors. This analysis may help uncover the TRPV channel-related genes associated with tumorigenesis. We also proposed novel strategies for tumor treatment.

## Introduction

The discovery of a new superfamily of channels called ‘transient receptor potential’ (TRP) channels has been described in the last two decades. These channels mediate calcium (Ca^2+^) ions entry into cells. Because Ca^2+^ ions play a central role in many cellular processes, including muscle contraction, cell proliferation, transmitter release, gene transcription, and cell death (1), studies investigating Ca^2+^ have increased markedly. For instance, TRP channels act as integrators of several significant signaling systems, including those mediated by cell surface receptors (such as G protein-coupled receptors and growth factor receptors), and mutations of TRP channel genes cause diseases in humans (2, 3). The TRP superfamily can be divided into seven subfamilies based on amino acid homology (4). Among all subfamilies, TRP vanilloid (TRPV) channels are the main players in nociception and integration of pain signals (5). The TRPV channel family currently comprises six members (TRPV1–6). Recent studies also showed that family members of TRPV channels play a vital important role in different cancers (6–9). For example, it has been reported that TRPV6 could activate nuclear factor of activated T cell (NFAT) transcription or phosphorylated Akt-signaling processes in prostate cancer (10). Overexpression of TRPV3 is correlated with tumor progression of non-small cell lung cancer (11), while breast cancer growth correlates with increased expression of TRPV6 (12). However, the genetics, epigenetic characteristics, and miRNA-mRNA interactions of TRPV channel-related genes are currently unclear across different cancer types.

In this study, we performed a pan-cancer analysis and comprehensively characterized of TRPV channel family members in 9125 tumor samples from 33 cancer types using multi-omics data from The Cancer Genome Atlas (TCGA) and the Drug Sensitivity in Cancer (GDSC) database and the Cancer Therapeutics Response Portal (CTRP) database. We also investigated the epigenetic regulation of the identified genes and revealed that changes in methylation status may lead to different prognostic outcomes. Furthermore, miRNA-mRNA analysis revealed the interaction network. Pathway activity analysis showed that TRPV channel-related genes were associated mainly with the inhibition of apoptosis, cell cycle, DNA damage response, and the AR hormone receptor. Importantly, by comprehensively analyzing two drug sensitivity databases, we predicted that TRPV channel-related genes are sensitive to targeted drugs. In conclusion, our study revealed the genomic alterations and clinical characteristics of members in TRPV channel-related genes.

## Materials and Methods

### Acquisition of Online Data Sets

To analyze the differential gene expression in normal tissues from healthy individuals, we used the Genotype-Tissue Expression (GTEx) dataset (version 7.0, https://commonfund.nih.gov/GTEx/). GTEx v.7 contains data from 714 postmortem donors and 11,688 RNA sequence data from 53 tissue sites, comprising 56,202 expression profiles of common genes. The large sample size and long follow-up time of the TCGA data set allow us to correlate genomic and transcriptomic profiles well to clinical outcomes and patient survival times. To perform a more detailed analysis, we retrieved raw data from TCGA (https://portal.gdc.cancer.gov/) and collected mRNA Seq data (n = 10,995), copy number variation (CNV) data (n = 11,495), single nucleotide variation (SNV) data (n = 8,663), methylation data (n = 10,129), and clinical data (n = 9,483). Reverse phase protein array (RPPA) data were downloaded from The Cancer Proteome Atlas (TCPA, https://tcpaportal.org/tcpa/index.html). The baseline expression of the TRPV channel-related genes was measured in 30 normal organs/tissues. Gene expression values of normal tissues were normalized by Transcripts per Million (TPM).

In the final pan-cancer analysis, samples from 33 types of cancer were investigated: adrenocortical carcinoma (ACC), acute myeloid leukemia (LAML), bladder urothelial carcinoma (BLCA), breast invasive carcinoma (BRCA), cervical squamous cell carcinoma and endocervical adenocarcinoma (CESC), colon adenocarcinoma (COAD), cholangiocarcinoma (CHOL), diffuse large B-cell lymphoma (DLBC), esophageal carcinoma (ESCA), glioblastoma multiforme (GBM), head and neck squamous cell carcinoma (HNSC), kidney renal clear cell carcinoma (KIRC), kidney renal papillary cell carcinoma (KIRP), kidney chromophobe (KICH), liver hepatocellular carcinoma (LIHC), lower grade glioma (LGG), lung adenocarcinoma (LUAD), lung squamous cell carcinoma (LUSC), mesothelioma (MESO), ovarian serous cystadenocarcinoma (OV), pheochromocytoma and paraganglioma (PCPG), pancreatic adenocarcinoma (PAAD), prostate adenocarcinoma (PRAD), rectum adenocarcinoma (READ), sarcoma (SARC), stomach adenocarcinoma (STAD), skin cutaneous melanoma (SKCM), testicular germ cell tumors (TGCT), thyroid carcinoma (THCA), thymoma (THYM), uterine corpus endometrial carcinoma (UCEC), uveal melanoma (UVM) and uterine carcinosarcoma (UCS).

The GDSC database (www.cancerrxgene.org) and the CTRP database (https://portals.broadinstitute.org/ctrp/) were used to investigate correlations between gene expression and drug sensitivity.

### Differential Gene Expression Analysis

We used RNA-Seq by Expectation-Maximization (RSEM) (13) to normalize the RNA-Seq data obtained from TCGA. Furthermore, paired TCGA mRNA expression values were represented as normalized RSEM values and samples with clinical information were included for further analyses. A total of 14 cancer types that harbored over 10 paired tumor and normal samples were included in the analysis. The fold change was represented as Tumor (mean)/Normal (mean), and the *P*-value was determined by the t-test, adjusted by the false discovery rate (FDR). To analyze the expression difference, the threshold values were determined as follows: fold change (FC) > 2, and adjusted *P*-value (FDR) < 0.05. If this threshold was not met, this type of cancer was excluded from the study.

### Subtype Analysis

Different cancer subtypes could be affected by different clinically relevant genes, such as in GBM, BRCA, LGG and DLBCL cancers. Furthermore, we performed an expression subtype analysis to identify specific marker genes for each cancer subtype. To make the analysis feasible, the number of cancer subgroups was set to at least 10, and we selected 6 cancer types for final analysis to identify relevant clinical genes. Subsequently, we analyzed genes relevant to TRPV-channel related genes using the Student’s t test (n_subtype = 2) and the ANNOVA t test (n_subtype>2), *P* < 0.05. The method used for clinically relevant analysis depended on the number of subgroups in each cancer subtype.

### Survival Analysis

mRNA expression and clinical survival data were combined by sample barcodes. For survival analysis, data consisting of 8,451 tumor samples from 25 cancer types (**Supplementary Table 1**) in the TCGA database, with more than 100 samples and a follow-up less than ten years for each cancer type were included. We used the median value of gene expression to divide tumor samples into “high” and “low” expression groups. The R package “survival” was used to fit the survival time and survival state of the two groups. The Cox proportional-hazards model for each gene was calculated using R. Genes with *P <*0.05 in the Kaplan-Meier logarithmic rank test were retained as significant.

### Single Nucleotide Variation Analysis

We collected SNV data (n = 8,663) of 33 cancer types from TCGA database. The downloaded data included the following variant types: Missense_Mutation, Silent, 5’ Flank, 3’ UTR, RNA, In_Frame_Del, Nonsense_Mutation, Splice_Site, Intron, 5’ UTR, In_Frame_Ins, Frame_Shift_Del, Nonstop_Mutation, 3’ Flank, Frame_Shift_Ins, and Translation_Start_Site. While samples with the Silent, Intron, IGR, 3’ UTR, 5’ UTR, 3’ Flank and 5’ Flank variants were excluded for the SNV percentage calculation with the R package “maftools” (14). The results are displayed in a waterfall plot. The percentage of SNVs in each gene coding region was calculated as the number of mutated samples divided by the number of cancer samples.

### Copy Number Variation Analysis

The raw data for the copy number variation (CNV) for 33 types of cancer types (n=11,495) were downloaded from TCGA database and analyzed using GISTICS2.0 (15). CNV was divided into two subtypes: heterozygous and homozygous, including amplification and deletion. Heterozygous variants, indicated a CNV occurring on one chromosome, while homozygous variants, defined variants on both chromosomes. To determine the CNV amplification and deletion percentages for the TRPV channel-related genes in each cancer, a percentage statistical analysis based on subtypes of CNV was performed using GISTIC2.0. Only genes with more than 5% CNV were considered a significant variation. With the method employed by Schlattl et al. (16), the association between paired mRNA expression and the percentage of CNV was calculated based on the Pearson’s product-moment correlation coefficient and the t distribution.

### Methylation Analysis

To investigate the methylation data of paired tumor and normal samples, we collected methylation data (n = 10,129) from TCGA database. Only 14 cancer types had normal-tumor paired data, and we further merged the mRNA expression data and methylation data using the sample barcode. To ensure the rigor of the data analysis, we calculated and included cancers with more than 10 normal-tumor paired by Pearson’s product-moment correlation coefficient (Student’s t-test, adjusted *P*-value<0.05). We merged overall survival data and methylation data.

Subsequently, the samples were divided into two groups by the median methylation status of the gene. Cox regression was performed to estimate the hazard ratio. Survival outcomes were estimated with the Kaplan-Meier method, and differences between survival distributions were evaluated by log-rank analysis with the ‘survival’ package in R software.

### MicroRNA Regulation Network Analysis

We collected microRNA (miRNA) expression data (n =9,105) of 33 cancer types from TCGA database. Subsequently, genes expression and miRNA expression were merged with TCGA sample barcodes. The Pearson’s correlation coefficient and t distribution were performed to calculate the association between paired mRNA and miRNA. After determining the adjusted *P*-value by FDR (cut-off values of FDR < 0.05 and R < 0), the correlation of all paired samples was calculated and the transcription factors were defined as positive regulators, the miRNA-gene pairs that had a negative correlation were considered as a potential negatively regulation pair. Finally, the regulation map was constructed using the “visNetwork” by R package.

### Pathway Activity Analysis

Based on the method of Rehan et al. (17), we calculated the scores of 7,876 samples from the TCPA (https://www.tcpaportal.org/) database of reverse phase protein array (RPPA). TCPA RPPA data was obtained from TCGA samples, and 32 cancer types and 10 cancer-related pathways were included. To obtain the relative protein levels, RPPA data of replicates-based normalization (RBN) were median-centered and normalized by the standard deviation. The score of each pathway activity was defined as the sum of the relative protein levels of all positive regulatory genes minus the sum of all relative protein levels of all negative regulatory genes according to the method by Ye et al. (18). We divided the tumor samples into two groups, high- and low- expression group, by the median expression of each gene. Subsequently, we performed a differences analysis of the pathway scores using Student’s t test, and adjusted the *P*-value by FDR, only FDR < 0.05 was considered as significantly affecting the pathway. When the pathway activity score of the X gene in the up-regulated group was greater than the score of the X gene in the down-regulated group, X was considered to have an activating effect on the pathway; otherwise, it had an inhibitory effect.

### Drug Sensitivity Analysis

CTRP (https://portals.broadinstitute.org/ctrp/) database version 2.0 contains 481 compounds and 860 cancer cell lines. The Genomics of Drug Sensitivity in Cancer (GDSC, https://www.cancerrxgene.org/) database includes over 1000 human cancer cell lines, drug response data, and genomic markers of sensitivity. We collected the IC50 of 265 small molecules in 860 cell lines and their corresponding mRNA gene expression from the GDSC. The mRNA expression data and drug sensitivity data were merged. Pearson’s correlation analysis was performed to determine the correlation between gene mRNA expression and the drug IC50. The *P*-value was adjusted using the FDR < 0.05. A Bubble plot was used to summarize the correlations between TRPV channel-related genes and drugs. Only a gene associated with at least one drug was selected. Only a drug associated with at least one gene was included in the analysis. Furthermore, we collected the IC50 of 481 small molecules tested in 1001 cell lines, and the corresponding mRNA gene expression from the CTRP. The mRNA expression data and drug sensitivity data were merged. Pearson correlation analysis was performed to get the correlation between gene mRNA expression and drug IC50. *P*-value was adjusted by FDR. By integrating correlation coefficient and FDR, only the top 30 ranked drugs were included.

### Plasmid Construction and Transfection

One optimal 21-mer short hairpin RNAs (shRNAs) targeting the human TRPV6 gene was used shRNA (CCTCTCCTTCTAGCTGCCAAA) (19). The shRNA lentiviral particles containing a scrambled shRNA sequence that did not lead to the specific degradation of any cellular mRNA was used as a negative control for the target shRNA lentiviral particles. The oligos and scrambled sequences were synthesized and cloned into the pure vector pLKO.1 following the Addgene protocol (http://www.addgene.org/tools/protocols/plko/), respectively. Lentiviruses were produced by cotransfection of a lentiviral plasmid, and packing the plasmids psPAX2 and pMD2.G into HEK-293T cells. After transfection into HEK-293T, the supernatant containing the virus was collected to infect cells. Stable cells infected with lentivirus were selected with puromycin and verified by RT-PCR.

### RNA Extraction and qRT-PCR

Total RNA was isolated from cells using Trizol reagent (Invitrogen, CA, USA) according to the manufacturer’s instructions and cDNA was synthesized using the PrimeScript RT Reagent Kit (TaKaRa, Shiga-ken, Japan) for qRT-PCR. Quantitation of all gene transcripts was performed by qRT-PCR using SYBR Green PCR Master Mix (TaKaRa, Shiga-ken, Japan), and GAPDH expression was used as an internal control. The primer pairs used are shown in **Supplementary Table 2**. Fold changes were calculated using the ΔΔCt method.

### Cell Culture and Reagents

MDA-MB-231, A549, and HEK-293T cell lines were tested by short tandem repeat (STR) in GENEWIZ (Suzhou, China) and cultured in DMEM medium supplemented with 10% fetal bovine serum in an incubator 5% CO_2_ at 37°C. All cell lines were negative for mycoplasma contamination. Lapatinib was purchased from SelleckChem.

### Scratch Assay

A total of 6×10^5^ cells were seeded in each well of a 6-well plate to form a monolayer overnight. A thin line was drawn in the middle of the cell monolayer with a 10 μL pipette tip. After washing with phosphate-buffered saline (PBS) twice, cells were cultured in DMEM with 2% FBS for 24 h in a 37°C incubator, and wounds were photographed at different time intervals. The distance of the wounds was measured using Photoshop software.

### Cell Proliferation Assays

The effect of lapatinib on the proliferation of different cancer cells was assayed using CCK-8 (US Everbright, Jiangsu, China) and 5-ethynyl-20 deoxyuridine (EdU) labelling assays according to the manufacturers’ instructions. Briefly, for the CCK8 assay, 1000 cells for each group were seeded in 96-well plates with 100 μL medium each well. After 24 hours of culture, 8 μM lapatinib was added to each plate. Each well was incubated with 10 μL CCK-8 solution for 2 hours and kept away from light before measuring the absorbance at 450 nm using the Thermo Scientific Multiscan plate reader (FC, 2011-06, USA). For the EdU labeling assay, cells were seeded on glass coverslips at 8×10^4^ cells per well in 12-well plates and incubated in DMEM containing 10% FBS with or without 8 μM lapatinib for 24 hours. EdU (Beyotime, Shanghai, China) was added to each well for 2 hours and cells were fixed and stained without exposure to light. Cells were counterstained with 4’,6-diamidino-2 phenylindole (DAPI) (nuclear staining) and then examined with a fluorescence microscope (Olympus) and photographed with a camera. The cell proliferation rate was assessed by calculating the proportion of EdU positive nucleus (red) to blue fluorescent nucleus by randomly counting 6 microscopic fields for each well in 5 separate experiments plates.

### Statistical Analysis

All statistical and computational analyses were performed using R version 4.0.3 (https://www.r-project.org/), and an unpaired Student’s t-test was used to compare two groups with normally distributed variables. The Kaplan-Meier method was used to estimate survival results, and the difference between survival distributions was evaluated by log-rank analysis using the “survival” package in the R software. The statistical significance cut-off value is set to *P <*0.05.

## Results

### Gene Expression and Subtype Analysis of TRPV Channel-Related Genes

**Figure 1:**
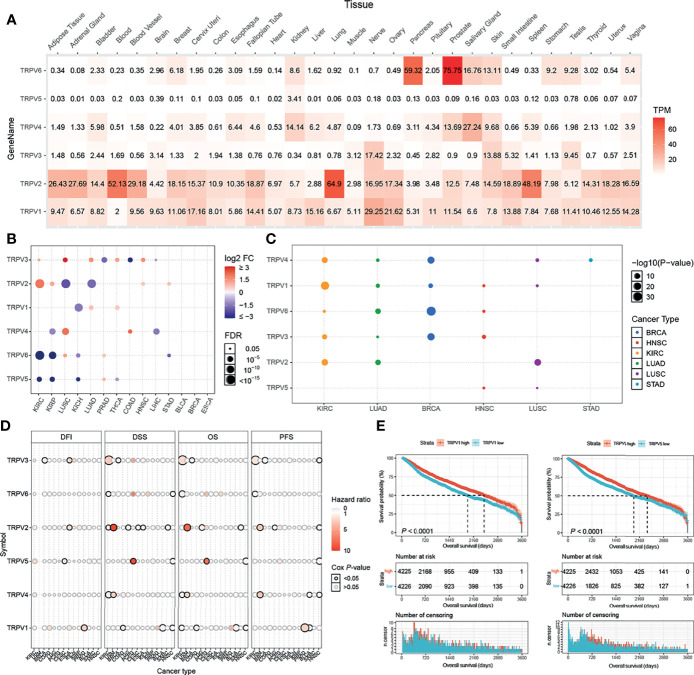
TRPV and subtype analysis of gene expression. **(A)** The heat map shows the expression profiles of the TRPV regulators in the GTEx dataset. **(B)** Significant differences in mRNA expression between normal and tumors in TCGA database. **(C)** Changes in mRNA expression in different cancer subtypes. **(D)** Survival analysis of TRPV regulators. The size of the dots represents the significance of the gene’s influence on survival for each cancer type,and the statistical significance of differences was determined by cox regression analysis. **(E)** TRPV1 and TRPV5 high expression contributes to the prognosis of cancer. All the significant differential expressed genes are shown in the figure.

TRPV channels belong to the subfamily of TRP channels (20). Six members of the TRPV family named TRPV1, TRPV2, TRPV3, TRPV4, TPRV5, and TRPV6 were identified and analyzed in our study. First, the differential expression of the TRPV channel-related genes was compared across normal tissues according to the GTEx database. TRPV6 was markedly up-regulated in the pancreas and prostate, while TRPV2 was overexpressed in the blood, lung, and spleen (**Figure 1A**). Subsequently, through TCGA expression data, we tested the differential expression of six members of the TRPV family in a pan-cancer analysis. Significant differential expression analysis between normal and tumor tissues showed that TRPV channel-related genes were abnormally expressed in 14 solid tumors. Significant differential data are shown in **Figure 1B**, and all data from differential expression analysis are shown in **Figure S1**. The heterogeneity of different cancers leads to differences in the expression of the TRPV channel family members. For example, TRPV5 and TRPV6 were down-regulated in most cancers, while TRPV2 and TRPV3 tend to be up-regulated in most cases. Analysis of the expression subtypes of regulators showed that different TRPV channel genes influence different caner subtypes. To identify clinically relevant genes that affect the cancer subtype, we performed an analysis of the expression each subtype of TRPV channel-related genes in different cancer subtypes. In BLCA and ESCA subtypes the expression of the TRPV channel-related gene did not show any significant differences. TRPV1 in KIRC, TRPV2 in LUSC, TRPV3 in BRCA, TRPV6 in LUAD and BRCA, while TRPV4 in KIRC and BRCA differed significantly across different cancer subtypes (*P <*0.05) (**Figure 1C**). Gene expression survival analysis showed that with regard to disease-free interval (DFI), high expression of TRPV3 in BRCA and ESCA, TRPV2 in ESCA, TRPV5 in CESC, and TRPV1 in PRAD, was associated with a longer prognosis. For disease-free survival (DSS), high expression of TRPV3 in KIRC, TRPV2 in UVM, and TRPV5 in PCPG was associated with a good prognosis. While for overall survival (OS), high expression of TRPV3 in KIRC, TRPV2 and TRPV4 in UVM, TRPV5 in PCPG led to prolonged survival, and for progression-free survival (PFS), high expression of TRPV3 in KIRC, TRPV2 and TRPV4 in UVM, TRPV1 in PRAD may be associated with a better survival (*P* < 0.05, **Figure 1D**).

Kaplan-Meier analysis showed that among all included samples with available clinical data, cases with high expression of TRPV1 and TRPV5 had a better prognosis (**Figure 1E**). TRPV2, TRPV4 and TRPV6 showed significant differences between the high and low expression group (*P* < 0.05, **Figure S2**).

### Analysis of the Somatic Mutation Profile of TRPV Regulators

**Figure 2:**
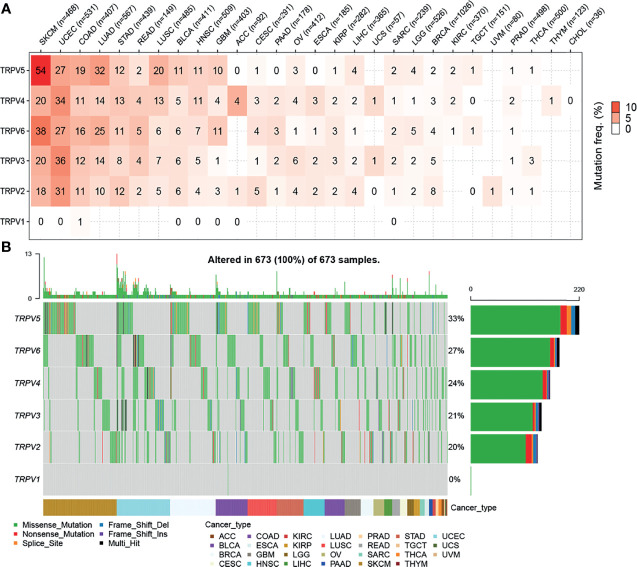
Single nucleotide variation (SNV) frequency and variant mutation types of TRPV channels. **(A)** Mutation frequency of TRPV channels. Numbers represent the number of percentages that have the corresponding mutated gene for a given cancer. The number ‘0’ indicates that there was no mutation in the gene coding region, and the blank space indicates that there was no mutation in any region of the gene. **(B)** SNV waterfall plot showing the mutation distribution of TRPV channels and a classification of variant SNV types.

First, we analyzed the mutation frequency of the different TRPV channel family members in a pan-cancer analysis. The SNV frequencies of each TRPV channel-related genes ranged 1%–54% among different cancer types. In SKCM, UCEC, COAD, and LUAD, except TRPV1, all members had a high mutation frequency ranging from 10%–54%. Meanwhile, TRPV1 showed only a 1% mutation frequency in COAD. Compared to other TRPV channel-related genes, TRPV1 had a lower mutation frequency among all cancer types (**Figure 2A**). A waterfall chart was used to analyze the specific mutation type. As shown in **Figure 2B**, the total SNV frequency of the TRPV channel-related genes was 100% (673 of 673 samples). The mutation frequencies of TRPV1, TRPV2, TRPV3, TRPV4, TRPV5, and TRPV6 were 0%, 20%, 21%, 24%, 33%, and 27%, respectively. In addition, the SNV frequency of the TRPV regulators was increased in SKCM, UCEC, LUAD, BLCA, and LUSC. TRPV5 had the highest mutation frequency of the TRPV family member genes, and SNV-associated survival analysis, determined that TRPV5 was tightly associated with differences in survival between mutated and normal genes in COAD samples (*P* < 0.05) (**Figure S3**).

### Copy Number Variation of TRPV Channel-Related Genes

**Figure 3:**
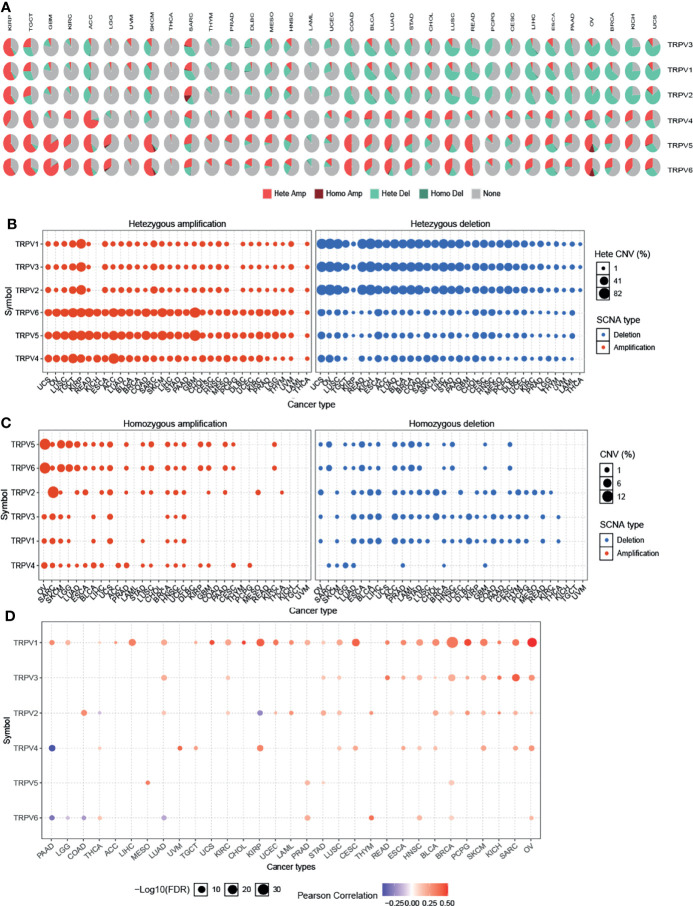
Copy number variation of TRPV channel-related genes. **(A)** CNV pie chart of 33 cancers. The CNV pie chart shows the combined heterozygous/homozygous CNV ratio of each regulator in each cancer. A pie chart representing the proportion of different types of CNV for each TRPV gene per cancer subtype; different colors represented different types of CNV. **(B)** The heterozygous CNV profile shows the percentage of heterozygous CNV, including the percentage of heterozygous amplification and deletion for each TRPV subtype gene in each cancer subtype. **(C)** The homozygous CNV profile shows the percentage of homozygous CNV, including the percentage of homozygous amplification and deletion for each TRPV subtype gene in each cancer. Only a gene with >5% CNV in a given cancer appears as a dot on the graph. **(D)** CNV correlation with TRPV channel mRNA expression. The Pearson product-moment correlation coefficient was used to study the association between CNV and mRNA expression. All dots with significant differences are shown. The size of a dot represents statistical significance, and the larger the size of the dot, the higher the statistical significance.

To identify CNV changes of TRPV channel-related genes at the chromosome level, we analyzed the CNV data from TCGA. As shown in **Figure 3A**, the pie chart including 33 cancer types showed that the main CNV of TRPV channel genes was a heterozygous amplification (Hete Amp) or deletion (Hete Del). In KIRP, except for TRPV4, all showed greater than 50% Hete Amp, while in UCS, TRPV1-3 presented more than 50% Hete Del. Subsequently, the CNV percentage analysis in specific cancer subtypes of Hete Amp and Hete Del showed that TRPV1-6 (except for TRPV4) in KIRP, TRPV4 in ACC, TRPV6 and TRPV5 in GBM had greater than 40% Hete Amp levels. Meanwhile, TRPV1-3 in OV and KICH exhibited greater than 80% Hete Del (*P <*0.05) (**Figure 3B**).

Furthermore, we analyzed the homozygous amplification (Homo Amp) and deletion (Homo Del). TRPV5 and TRPV6 in OV and TRPV2 in SARC subtypes had greater than 10% Homo Amp, while TRPV5 and TRPV6 in LAML, TRPV2, and TRPV3 in CHDL had greater than 5% Homo Del levels (*P <*0.05) (**Figure 3C**). The Pearson’s product-moment correlation coefficient was used to study the association between mRNA expression and CNV. The correlation analysis indicated that mRNA expression was positively correlated with CNV. For example, TRPV1 mRNA expression was positively correlated with CNV, especially in hormone-dependent tumors, such as BRCA and OV for which this correlation was more significant (*P <*0.05). However, there was a negative correlation between TRPV6 in PAAD, LGG, COAD, and LUAD (*P <*0.05, **Figure 3D**). These results suggested that the CNV of TRPV channel-related genes could mediate their abnormal expression of mRNA, which may play an important role in cancer progression.

### Methylation Analysis of TRPV Channel-Related Genes

**Figure 4:**
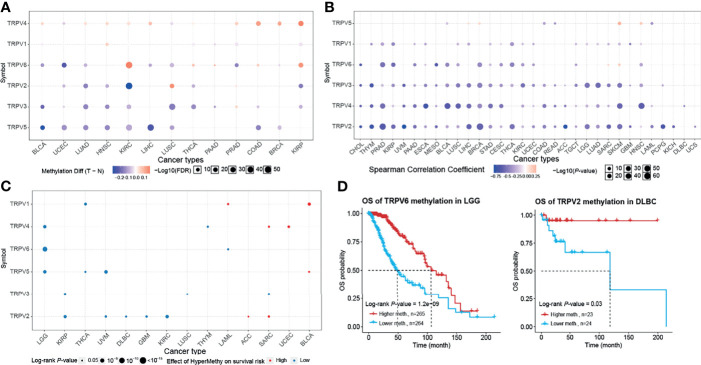
Methylation and survival of each TRPV channel-related genes. **(A)** Differential methylation in TRPV channels between tumor (T) and normal (N) samples in each cancer. Red dots represent increased methylation in tumors and blue dots represent decreased methylation in tumors. The darker the dot color, the larger the difference in methylation level. **(B)** Correlation between methylation and mRNA gene expression. Red points represent a positive correlation, and blue dots represent a negative correlation. The darker the dot color means the stronger the correlation. **(C)** Survival difference between TRPV regulators with high and low methylation levels and samples. Red dots represent worse survival of the hypermethylation group; blue dots represent the opposite. The dot size represents the statistical significance, the larger the dot size means, the higher the statistical significance. **(D)** Prognosis analysis of TRPV6 methylation status in brain lower grade glioma (LGG) and TRPV2 methylation in the lymphoid neoplasm, diffuse large B-cell lymphoma (DLBC). *P <* 0.05; FDR, false discovery rate.

Epigenetic modification plays an important role in tumor progression (21–23). Hence, we performed an evaluation of the methylation status of TRPV channel-related genes to identify potential differences epigenetic regulation. There were more hypermethylated genes than hypomethylated genes in COAD and KIRP cancers; whereas there were more hypomethylated than hypermethylated genes in BLCA, UCEC, LUAD, HNSC, KIRC, LIHC, LUSC, THCA, and BRCA subtypes. TRPV3 and TRPV5 were hypomethylated in most cancer types, while TRPV4 was hypermethylated (*P <*0.05) (**Figure 4A**). The methylation level of TRPV channel-related genes has a difference among cancer types. Furthermore, we evaluated TRPV channel-related genes methylation and mRNA expression through Pearson’s correlation analysis. The results indicated that most mRNA expression levels of the TRPV channel-related genes were negatively correlated with their degree of methylation. However, the methylation levels of TRPV5 and TRPV6 had a positive correlation with mRNA expression in SKCM (*P <*0.05) (**Figure 4B**). Kaplan-Meier survival analysis indicated that the hypermethylation of TRPV1 in LAML and BLCA, TRPV4 in SARC and UCEC, TRPV5 in BLCA, TRPV2 in ACC, and SARC cancers was associated with a poor prognosis in most tumors. Meanwhile, the hypomethylation of TRPV4, TRPV5, and TRPV6 in LGG, TRPV2 in KIRP, UVM, DLBC, GBM, and KIRC was associated with a poor prognosis (*P <*0.05) (**Figure 4C**). Further survival analysis indicated that hypomethylation of TRPV6 in LGG and hypomethylation of TRPV2 in DLBC were associated with a poor prognosis (*P <*0.05) (**Figure 4D**).

### Relevant miRNA Regulation Analysis

**Figure 5:**
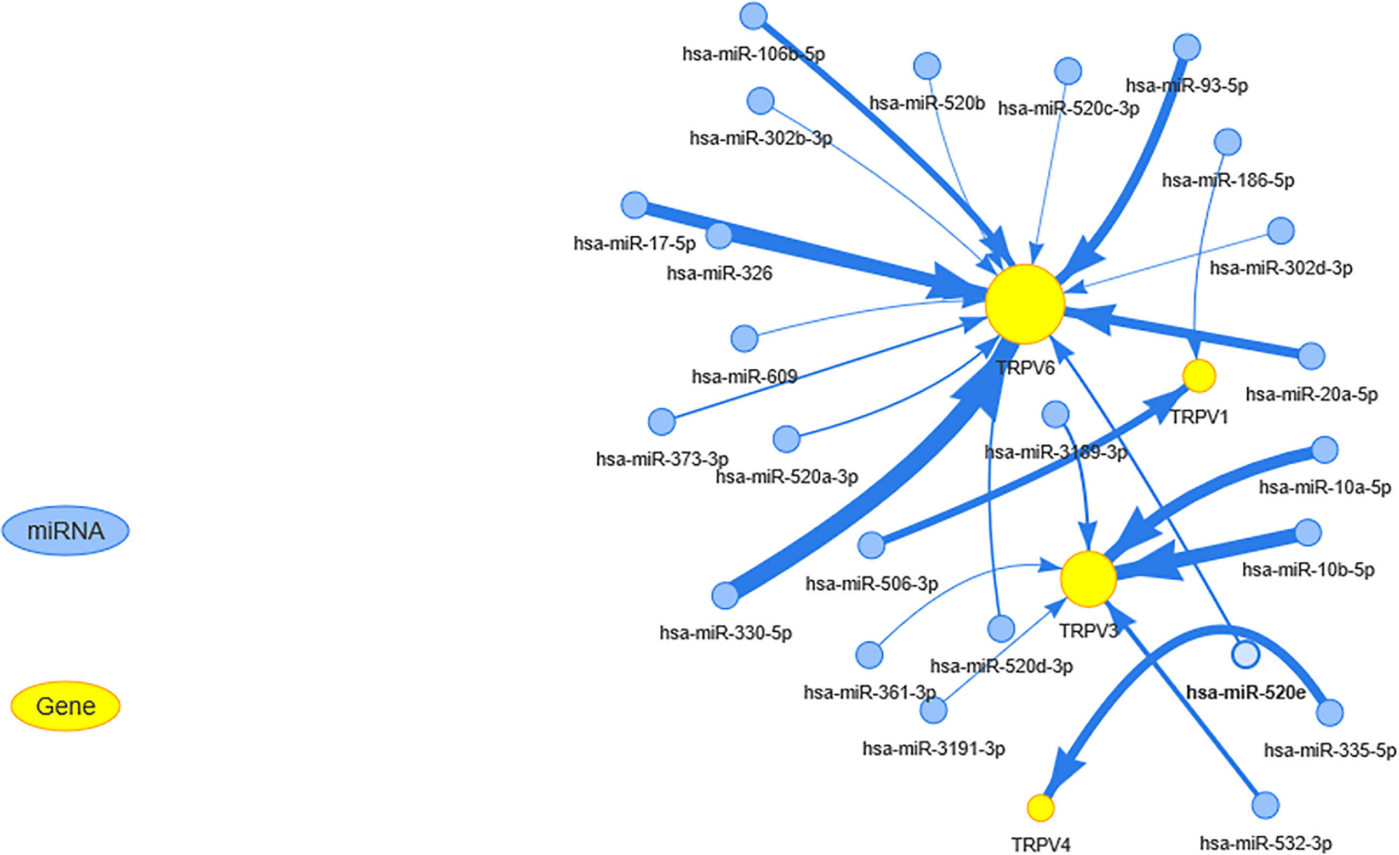
The miRNA regulation network of TRPV channel-related genes. The connection between mRNA and miRNA indicates that the mRNA is regulated by this miRNA. The larger the yellow dot size, the greater the association with miRNA regulation. The size of arrow edge width depends on the absolute value of the correlation coefficient.

miRNA regulates mRNA expression and may play a vital role in cancer (24, 25). To determine whether miRNA could regulate the TRPV channel family gene expression, we used visNetwork to construct miRNA gene regulation networks. **Figure 5** shows that miRNAs may regulate the expression of mRNA of TRPV channel-related genes by targeting TRPV1, TRPV3, TRPV4, and TRPV6. In particular, TRPV6 could be down-regulated by a higher number of miRNAs, including hsa-miR-330-5p, hsa-miR-93-5p, and hsa-miR-17-5p. Instead, hsa-miR-10a-5p and hsa-miR-10b-5p could negatively regulate TRPV3 expression. These results indicated that TRPV channel expression may be regulated by miRNA and may affect cancer progression.

### Pathway Activity Analysis

**Figure 6:**
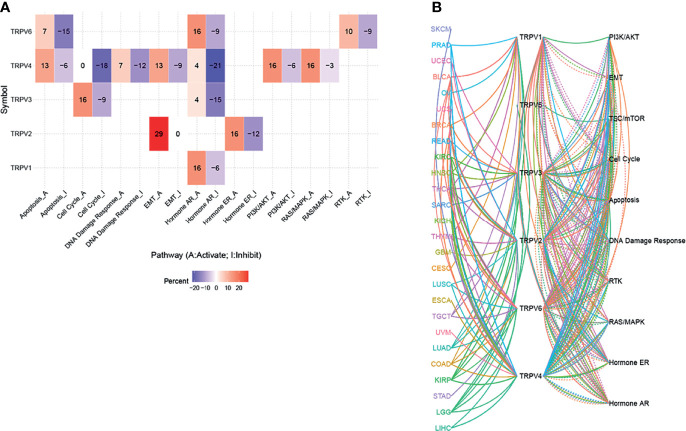
The pathway activity network between TRPV channels. **(A)** The combined percentage of TRPV regulator genes influencing pathway activity. **(B)** The line represents the connection between different pathways, where solid lines of the connecting pathways represent activation and dotted lines of the connecting pathways represent inhibition. The colors of the lines represent different types of cancer.

From the pathway relation network, shown in **Figure 6A**, TRPV channel-related genes were involved in apoptosis, cell cycle, DNA damage response, epithelial-mesenchymal transition (EMT), the hormone receptors AR and ER, and the PI3K/AKT, RAS/MAPK, and RTK signaling pathways. TRPV6 was involved mainly in the inhibition of apoptosis (15% inhibition versus 7% activation), while TRPV4 showed the opposite results in the apoptosis pathway (13% activation versus 6% inhibition). TRPV4 also inhibited the cell cycle pathway (18%) and the DNA damage response (12%), while TRPV3 showed the opposite function in the cell cycle pathway (16% activation versus 9% inhibition). TRPV2 exhibited a strong activation function in EMT with 29% activation. With regard to hormone AR levels, TRPV6, TRPV4, TRPV3, and TRPV1 was associated with 9%, 21%, 15%, 6% inhibition, while AR expression was activated by 16%, 4%, 4%, 16%, respectively. TRPV4 was also involved in the activation of the PI3K/AKT pathway (6% inhibition versus 16% activation) and the RAS/MAPK pathway (3% inhibition versus 16% activation). For the RTK pathway, TRPV6 was associated with 10% activation of the pathway (*P <*0.05) (**Figure 6A**). The pathway relationship network analysis revealed that the pathways corresponding to each TRPV channel and the corresponding cancer (**Figure 6B**). **Figure S4** shows a pie chart of pathway activation and pathway inhibition of different molecules related to the TRPV channel genes.

### Drug Sensitivity Analysis

**Figure 7:**
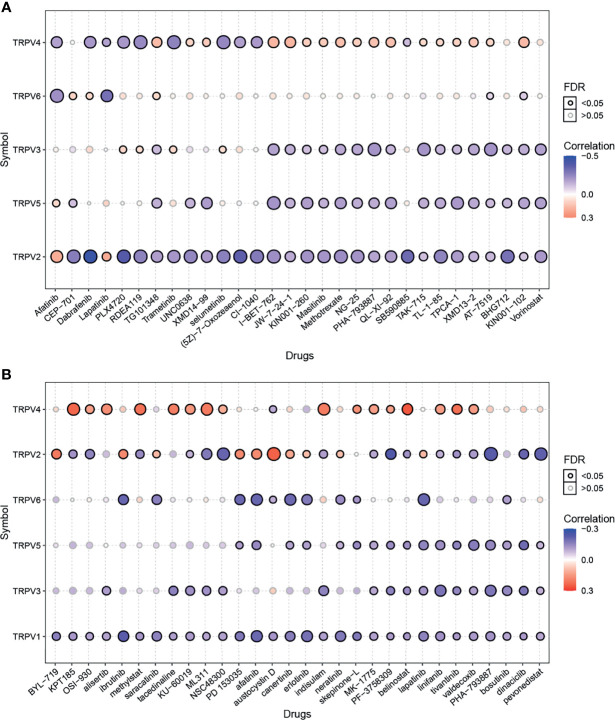
Drug sensitivity analysis of TRPV channel-related genes. **(A)** The gene set drug sensitivity analysis from Genomics of Drug Sensitivity in Cancer (GDSC) IC50 drug data. **(B)** The gene set drug sensitivity analysis from Cancer Therapeutics Response Portal (CTRP) IC50 drug data. The Pearson’s correlation indicates the correlation between gene expression and drugs sensitivity. Blue bubbles represented negative correlations, and red bubbles represented positive correlations; the deeper the color, the higher the correlation. The bubble size was positively correlated with the FDR significance. The black outline indicates an FDR < 0.05. Only the top 30 ranked drugs were included.

Genomic alterations influence the clinical response to chemotherapy and targeted therapy (26). To investigate the role of TRPV channel-related genes in chemotherapy or targeted therapy, we integrated drug sensitivity and gene expression profile data from GDSC and CTRP cancer cell lines. We performed Pearson’s correlation analysis, the results showed that in the GDSC database, drug sensitivity to dabrafenib, PLX4720, RDEA119, trametinib, selumetinib, (5Z)-7-oxozeaenol, CI-1040, SB590885 was negatively correlated with TRPV2 and TRPV4 expression according to the IC50 value. Although drug resistance toward afatinib and lapatinib was positively associated with TRPV2 expression, TG101348, UNC0638, XMD14-99 I-BET-762, JW-7-24-1, KIN001-260, masitinib, methotrexate, NG-25, PHA-793887, QL-XI-92, TAK-715, TL-1-85, TPCA-1, XMD13-2, AT-7519, and KIN001-102 were positively correlated with TRPV4 expression (**Figure 7A**). In the CTRP database, the drug sensitivity to KPT185, OSI-930, methylstat, KU-60019, ML311, NSC48300, indisulam, MK-1775, PF-3758309, belinostat, linifanib, tivantinib, valdecoxib, PHA-793887, dinaciclib, and pevonedistat was negatively correlated with the expression of TRPV1 and TRPV2 and with the IC50 value. However, with drug resistance analysis, most of these drugs were positively associated with TRPV4 expression based on the IC50 (**Figure 7B**).

### Lapatinib and TRPV6 Regulated Cancer Cell Proliferation

**Figure 8:**
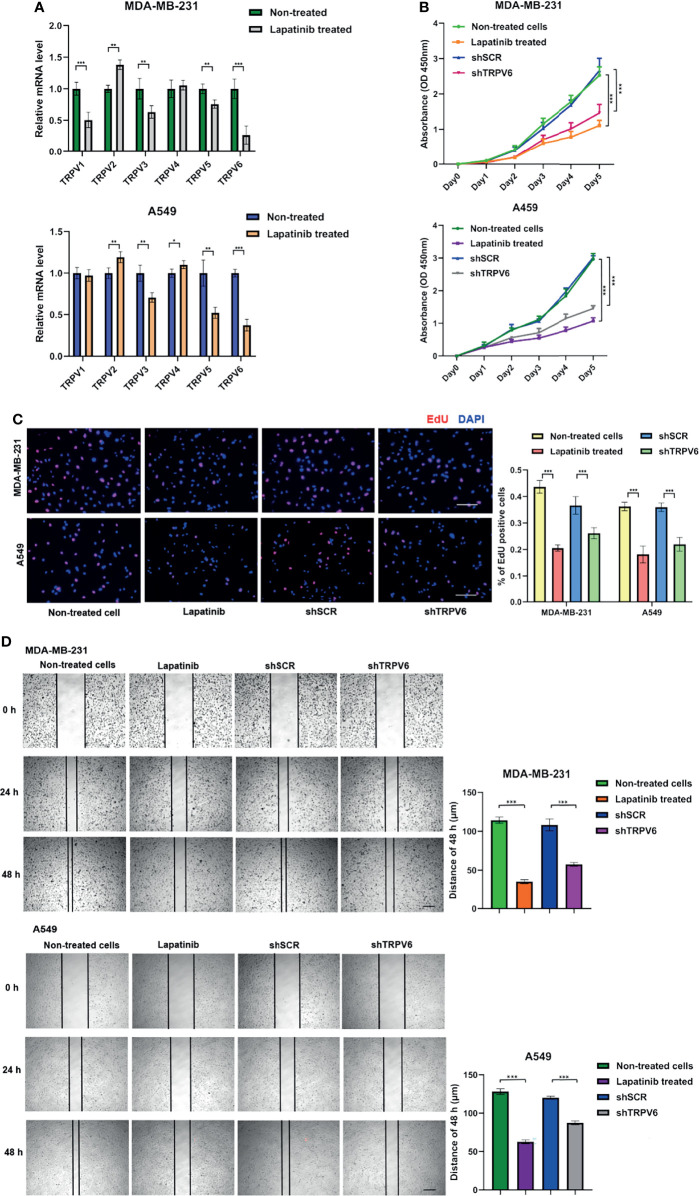
Effects of lapatinib and TRPV6 knockdown on cell proliferation and apoptosis in cancer cells. **(A)** TRPV channels mRNA expression in MDA-MB-231 and A549 cell lines after treatment with lapatinib. **(B)** The effect of TRPV6 knockdown and exposure to lapatinib on cell proliferation in MDA-MB-231 and A549 cell lines examined by the CCK8 assay and the **(C)** EdU assay. **(D)** The scratch assay was performed in MDA-MB-231 and A549 cells treated with shTRPV6 or 8 μM lapatinib. Values were expressed as mean ± SD from three independent experiments (Student’s t test, *P < 0.05, **P < 0.01, ***P < 0.001). Scale bars, 50 μm.

We showed that TRPV6 may play a role in the promotion of cancer development in breast cancer and lung adenocarcinoma, and lapatinib is negatively correlated with the expression of most TRPV molecules. To verify the reliability of the results, we performed functional studies including two types of cancer. Lapatinib is a tyrosine kinase inhibitor (TKIs). We investigated the effects of exposure to lapatinib on the expression of TRPV family genes using a qPCR assay (**Figure 8A**). The results showed that lapatinib could inhibit the expression of TRPV3, TRPV5, and TRPV6 in both MDA-MB-231 cells and A549 cells. The reduction in mRNA expression of TRPV6 was the most obvious after exposure to lapatinib. To study whether the TRPV6 channel is involved in regulating cell proliferation and migration in breast cancer and lung adenocarcinoma cells, shRNA targeting the TRPV6 gene was used to silence TRPV6 expression (**Figure S5**). The CCK8 assay and the EdU assay showed that the reduction of TRPV6 and the application of lapatinib could lead to lower cell proliferation of MDA-MB-231 and A549 cell lines (**Figures 8B, C**). The migration assay showed that TRPV6 reduction and lapatinib application could lead to lower cell migration capacity than their control groups, respectively, suggesting that inhibition and reduction of TRPV channel-related genes could inhibit cell migration (**Figure 8D**).

## Discussion

Since the channels of TRPV were discovered two decades ago, researches have focused on their role in the regulation of ion channels (4, 27, 28). However, in recent years, an increasing number of studies have reported that TRPV channel-related genes play an important role in carcinogenesis (5, 28–30). Hence, research focusing on TRPV channel-related genes in tumorigenesis and exploring the potential targets of clinical treatment are of vital importance. In our study, we performed a systematic characterization and comprehensive analysis of TRPV channel-related genes across 33 types of cancer by mining multi-omics profiling data. Our results shed light on the overall role of TRPV channel-related genes in cancer by not only revealing multiple potential mechanisms of TRPV regulators in different cancer contexts, but also identifying TRPV regulators associated with cancer pathways.

In this study, we focused on 6 TRPV channel-related proteins, including TRPV acting as cell-autonomous mediators and Ca^2+^ signaling mediators (31). The over-expression of TRPV6 mRNA has been reported in breast cancer (32). However, the genomic characteristics of other TRPV channel-related genes have not been described in a pan-cancer analysis. Our results showed that TRPV channel-related genes are dysregulated in different cancers types, such as KIRC, KIRP, LUSC, and LUAD. Meanwhile, through genetic analysis, we also found that there is a high frequency of SNV and CNA among TRPV channel-related genes. CNA are positively associated with mRNA expression (33), and may cause the gene to become an oncogene especially with regard to the sodium and calcium regulators TRPV1 and TRPV2. Peters et al. reported that the differential expression of TRPV6 in breast cancer cells and tumor sections is most likely a result of gene amplification (34), which is in agreement with the results of our study. The epigenetic modification analysis of each regulatory gene shows that abnormal hypermethylation of TRPV channel-related genes mediated their down-regulation and was associated with a poor prognosis in several cancers. Hypermethylation and survival analysis for TRPV6 in LGG and TRPV2 in DLBC, suggested that the hypermethylation status may be drivers in those cancers. The same trend was observed for TRPV4 and TRPV5 in LGG, TRPV2 in KIRP, and KIRC. Therefore, we considered that genetic and epigenetic modification alterations of TRPV channel regulatory genes can lead to dysfunction and, in some cases, be involved in tumorigenesis. Since TRPV channel-related genes play such an important role in cancer, identifying their regulatory molecules is crucial. Through the miRNA-mRNA interaction network, we determined that TRPV1, TRPV3, TRPV4, and TRPV6 could be regulated by miRNAs. Zhou et al. (35) reported that translational TRPV1 could be down-regulated by miR-199 to regulate visceral pain in patients. Our results indicated that TRPV could also be influenced by other miRNAs and treatment targets were also identified.

Herein, we reveal the potential role of TRPV channel-related genes in cancer, and the targeted drug-sensitivity analysis for these molecules was also predicted using available databases. TRPV channels are closely related to pain (36, 37), and the results of our study imply these channels may play a leading role in tumor analgesia. Although the biological correlation, cannot be compared with the same degree as the statistical correlation, some correlations were greater than 0.3, but for the presentation of the results, we set the threshold to 0.3 (**Figure 7**). Nevertheless, the potential mechanisms of the drug’s effects on TRPV channel-related genes expression and cancer progression require further investigation. Since it has been reported that TRPV channels play an important role among cancers, we also performed experiments that validate the role of TRPV channel-related genes in tumor proliferation and cell migration.

## Conclusion

In this study, we compared the gene expression profiles of TRPV channel-related genes in tumors and in the corresponding normal tissues. We showed that the SNV and CNV alternations of the genome had an impact on mRNA levels and survival. Furthermore, accessible databases were used to predict that TRPV channel-related genes that can be regulated by miRNA and to identify targeted drugs for TRPV molecules. Finally, verification of these findings *in vitro* using cancer cell lines also supports the findings of our study.

## Data Availability Statement

The datasets presented in this study can be found in online repositories. The names of the repository/repositories and accession number(s) can be found in the article/**Supplementary Material**.

## Author Contributions

HZ, XXW, and GL contributed to the conception and design of the study. GL and XXW performed the research and data analysis. XXW and YZ wrote the first draft of the manuscript. XXW, XHW, and QL wrote sections of the manuscript. LL, LQ, and ZQ were responsible for collecting online clinical information. SZ provided the essential reagents. All authors listed have made a substantial, direct, and intellectual contribution to the work and have approved the study for publication.

## Funding

This work was supported by the National Natural Science Foundation of China (grant NO. 81770213), Natural Science Foundation of Tianjin (grants NO. 19JCYBJC26500 and 18JCZDJC45100).

## Conflict of Interest

The authors declare that the research was conducted in the absence of any commercial or financial relationships that could be construed as a potential conflict of interest.

## Publisher’s Note

All claims expressed in this article are solely those of the authors and do not necessarily represent those of their affiliated organizations, or those of the publisher, the editors and the reviewers. Any product that may be evaluated in this article, or claim that may be made by its manufacturer, is not guaranteed or endorsed by the publisher.

## Supplementary Material

Supplementary Figure 1 Differential mRNA expression of all data between paired normal and tumor samples in the TCGA database. Significant differences data are color-labeled, while genes without significant differences are in transparent color.

Supplementary Figure 2 Survival analysis of TRPV2, TRPV3, TRPV4, and TRPV6.

Supplementary Figure 3 Overall survival associated with mutations of TRPV5 channel-related regulators across cancer subtypes.

Supplementary Figure 4 The percentage of pathway activation and inhibition of TRPV channel regulators.

Supplementary Figure 5 Evaluation of TRPV6 mRNA expression in MDA-MB-231 and A549 cells treated with shRNA (Student’s t test, ****P <*0.001).
