# Supplementary figures and images for: Pan-Cancer Analysis Reveals Genomic and Clinical Characteristics of TRPV Channel-Related Genes
Source: Front Oncol. 2022 Jan 31;12:813100. doi: 10.3389/fonc.2022.813100 (PMC8841404; doi:10.3389/fonc.2022.813100)

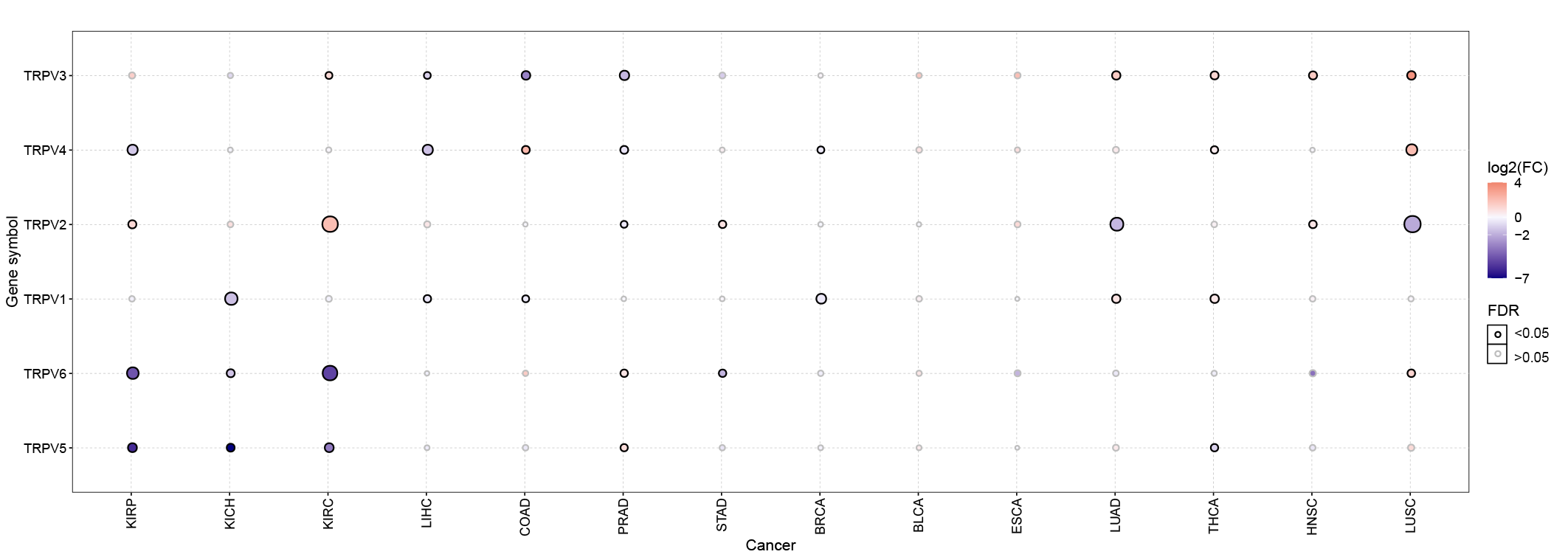

Supplement: Supplementary Figure 1 — Differential mRNA expression of all data between paired normal and tumor samples in the TCGA database. Significant differences data are color-labeled, while genes without significant differences are in transparent color. [file Image_1.tif]

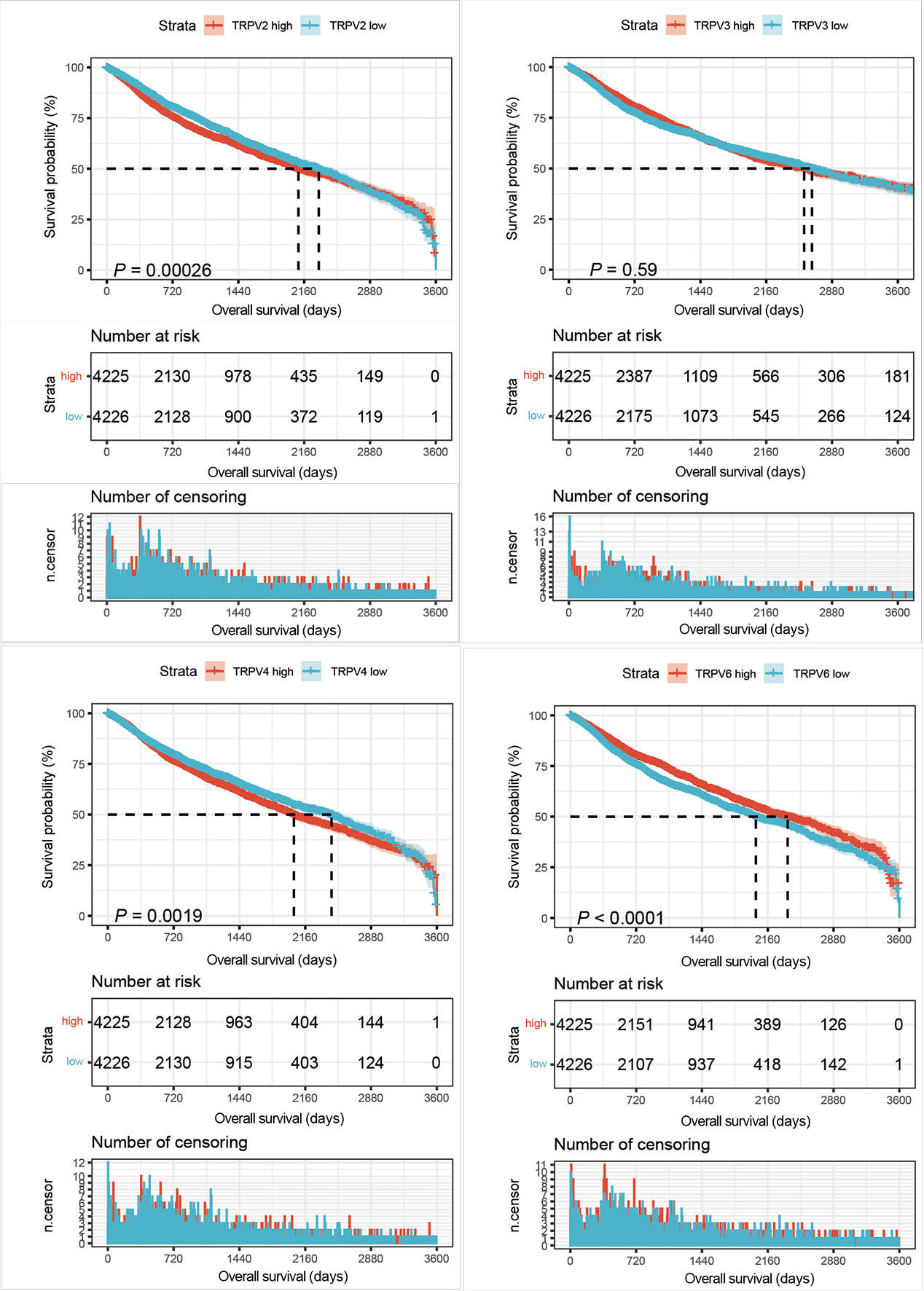

Supplement: Supplementary Figure 2 — Survival analysis of TRPV2, TRPV3, TRPV4, and TRPV6. [file Image_2.tif]

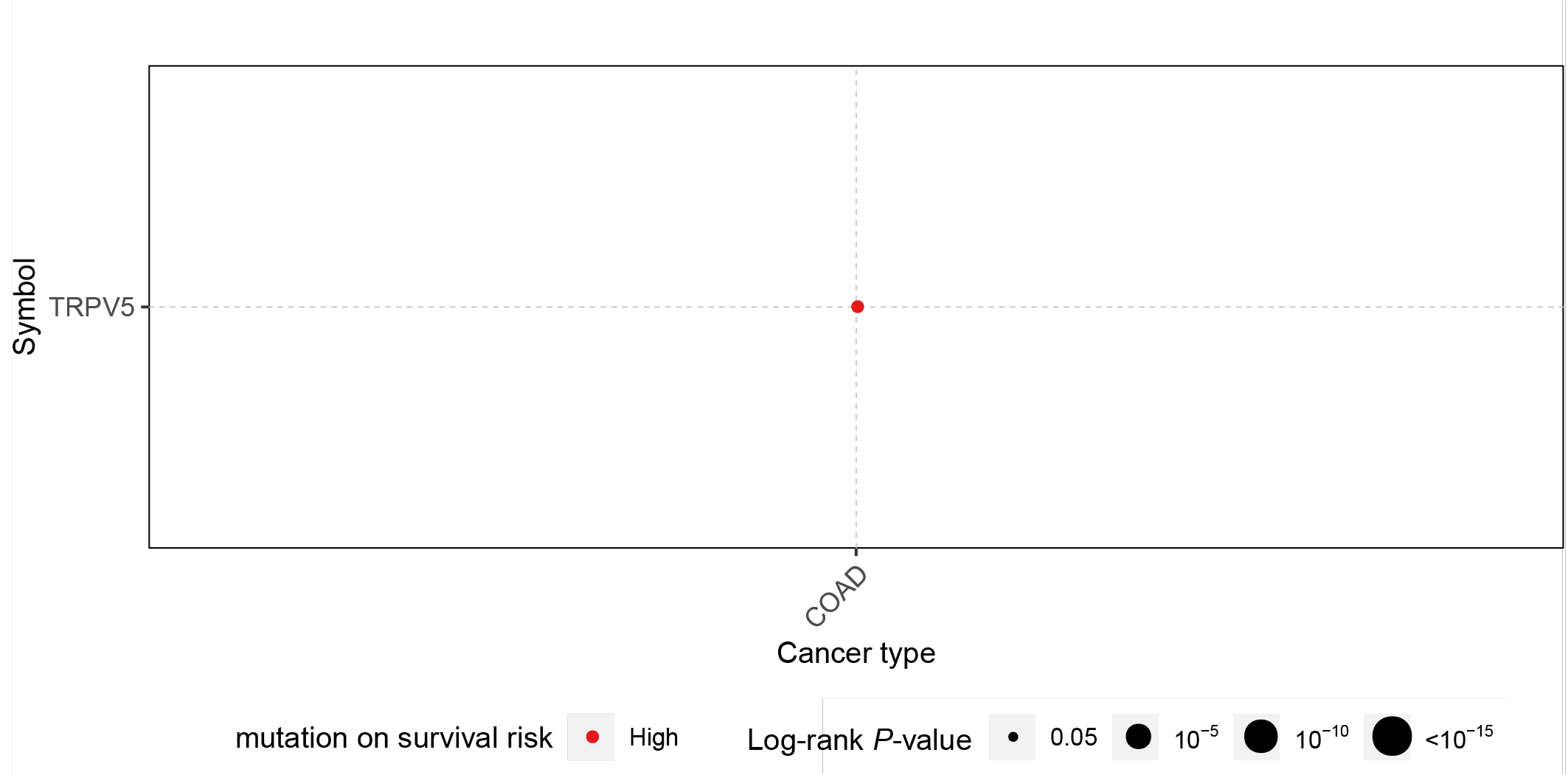

Supplement: Supplementary Figure 3 — Overall survival associated with mutations of TRPV5 channel-related regulators across cancer subtypes. [file Image_3.tif]

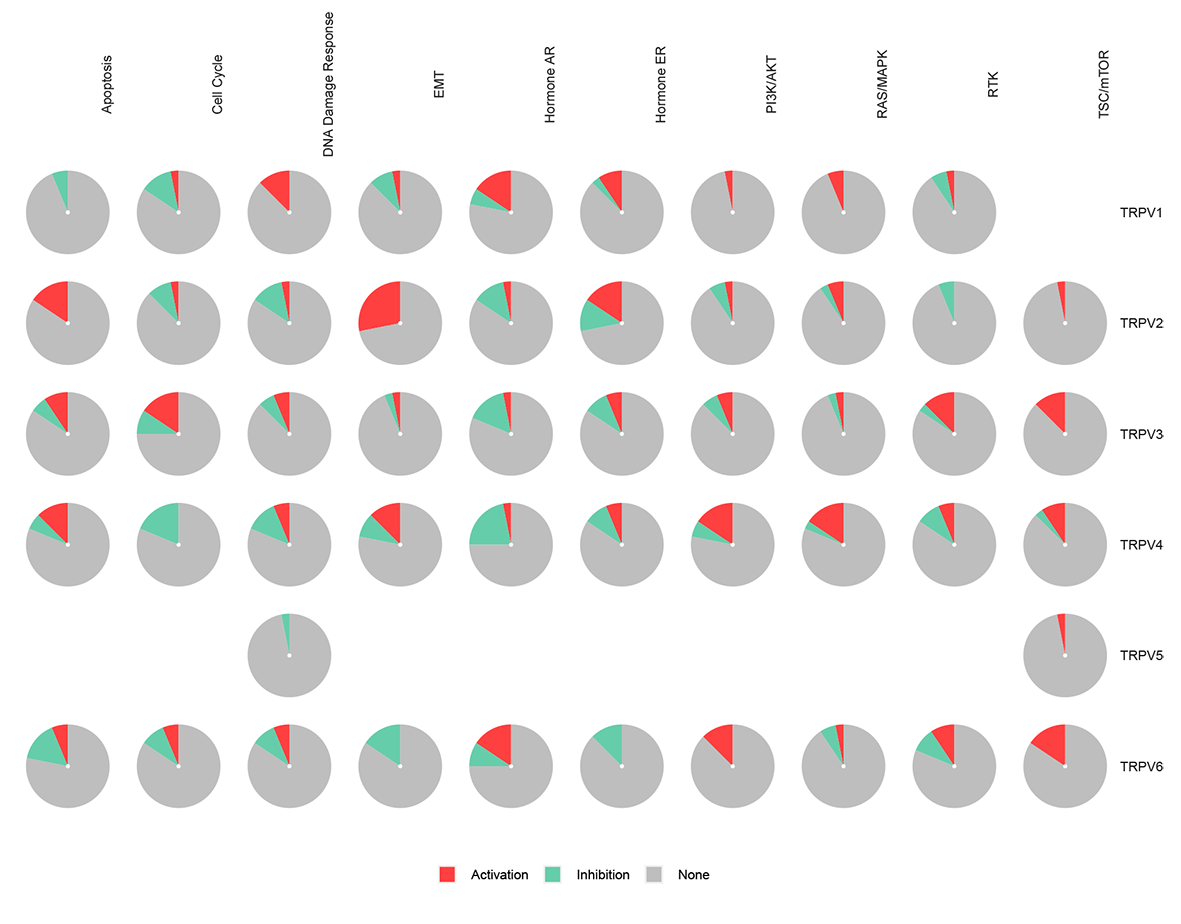

Supplement: Supplementary Figure 4 — The percentage of pathway activation and inhibition of TRPV channel regulators. [file Image_4.tif]

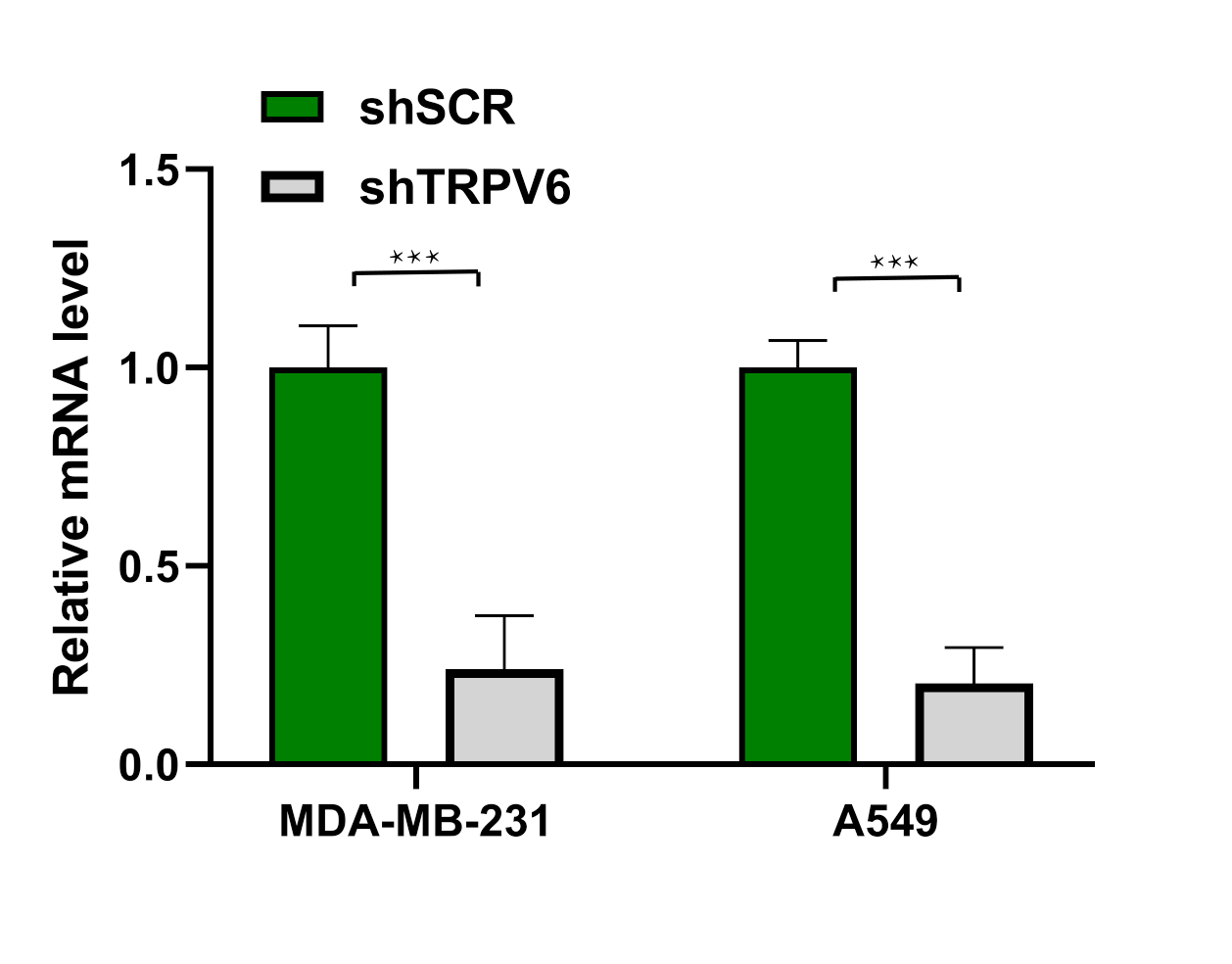

Supplement: Supplementary Figure 5 — Evaluation of TRPV6 mRNA expression in MDA-MB-231 and A549 cells treated with shRNA (Student’s t test, ***P <0.001). [file Image_5.tif]
